# Simultaneous Release of Silver Ions and 10–Undecenoic Acid from Silver Iron–Oxide Nanoparticles Impregnated Membranes

**DOI:** 10.3390/membranes12060557

**Published:** 2022-05-27

**Authors:** Gheorghe Nechifor, Alexandra Raluca Grosu, Andreea Ferencz (Dinu), Szidonia-Katalin Tanczos, Alexandru Goran, Vlad-Alexandru Grosu, Simona Gabriela Bungău, Florentina Mihaela Păncescu, Paul Constantin Albu, Aurelia Cristina Nechifor

**Affiliations:** 1 Analytical Chemistry and Environmental Engineering Department, University Politehnica of Bucharest, 011061 Bucharest, Romania; ghnechifor@gmail.com (G.N.); andra.grosu@upb.ro (A.R.G.); andra_d24@yahoo.com (A.F.); alexandru@santego.ro (A.G.); florynicorici@yahoo.com (F.M.P.); aureliacristinanechifor@gmail.com (A.C.N.); 2 Department of Bioengineering, University Sapientia of Miercurea-Ciuc, 500104 Miercurea-Ciuc, Romania; tczszidonia@yahoo.com; 3 Department of Electronic Technology and Reliability, Faculty of Electronics, Telecommunications and Information Technology, University Politehnica of Bucharest, 061071 Bucharest, Romania; 4 Department of Pharmacy, Faculty of Medicine and Pharmacy, University of Oradea, 410028 Oradea, Romania; sbungau@uoradea.ro; 5 Radioisotopes and Radiation Metrology Department (DRMR), IFIN Horia Hulubei, 023465 Măgurele, Romania

**Keywords:** control release, composite membranes, impregnated membranes, silver–iron oxide nanoparticles, silver ions, 10–undecenoic acid, cellulose derivatives, cellulose acetate, polysulfone

## Abstract

The bio-medical benefits of silver ions and 10–undecenoic acid in various chemical-pharmaceutical preparations are indisputable, thus justifying numerous research studies on delayed and/or controlled release. This paper presents the effect of the polymer matrix in the simultaneous release of silver ions and 10–undecenoic acid in an aqueous medium of controlled pH and ionic strength. The study took into consideration polymeric matrices consisting of cellulose acetate (CA) and polysulfone (PSf), which were impregnated with oxide nanoparticles containing silver and 10–undecenoic acid. The studied oxide nanoparticles are nanoparticles of iron and silver oxides obtained by an accessible electrochemical method. The obtained results show that silver can be released, simultaneously with 10–undecenoic acid, from an impregnated polymeric membrane, at concentrations that ensure the biocidal and fungicidal capacity. Concentrations of active substances can be controlled by choosing the polymer matrix or, in some cases, by changing the pH of the target medium. In the studied case, higher concentrations of silver ions are released from the polysulfone matrix, while higher concentrations of 10–undecenoic acid are released from the cellulose acetate matrix. The results of the study show that a correlation can be established between the two released target substances, which is dependent on the solubility of the organic compound in the aqueous medium and the interaction of this compound with the silver ions. The ability of 10–undecenoic acid to interact with the silver ion, both through the carboxyl and alkene groups, contributes to the increase in the content of the silver ions transported in the aqueous medium.

## 1. Introduction

Controlled, delayed or directed release of various chemical species with chemical, biochemical or biological activity is one of the most important applications of membranes [1,2]. They are released under control, through or from membranes, in specific environments: drugs, pharmaceuticals and phytopharmaceuticals, detergents and dyes, fertilizers, nutrients, pesticides, fungicides or herbicides with technical, economic and environmental benefits [3,4,5].

**Figure 1:**
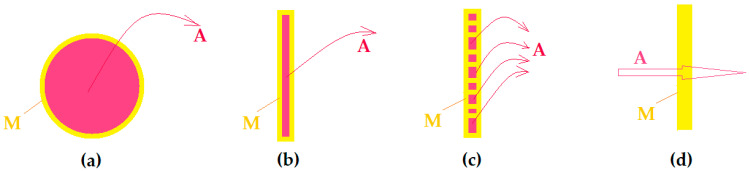
Schematic representation of releasing of an active species (A): from (**a**–**c**); or through (**d**); a membrane (M).

In all these cases, the membrane (M) is the one that releases, in a controlled, delayed or directed way, the active chemical species (A), which it incorporates in a dedicated target environment (Figure 1).

The immobilized target chemical species can be released from inside polymer capsules or boxes (spheres) (Figure 1a) from two selectively permeable polymer films (Figure 1b) or from the pores of a polymer matrix (Figure 1c). On the other hand (Figure 1d), any membrane barrier can be interposed between the source phase containing the target chemical species and a receiving phase (this is the case of various devices that use dialysis-type membrane).

This technique of administration of an active chemical species leads to the reduction of the consumption of valuable active substances, the dosage at a constant and controllable level, the prolongation of the duration of action, the avoidance of overdose and the decrease in the impact on the environment [6,7,8].

Among the chemical species of interest for controlled release, silver ions [9] and 10–undecenoic acid [10] have attracted the attention of researchers.

In particular, silver nanoparticles have been extensively studied [11,12,13,14,15] for their biocidal (bactericidal) action, being known and accepted three mechanisms of action on cells (bacteria):

- Attachment to the surface cell membrane of the silver nanoparticle with dimensions below 10 nm, leading to disturbance of respiration and/or cell permeability [16];
- Penetration of the cell membrane, having the effect of blocking the functions containing sulfur or phosphorus [17];
- The release of silver ions by the nanoparticle, amplifying its local effect [18].

On the other hand, the fungicidal action of 10–undecenoic acid has been the subject of research [19,20,21] because it is a very accessible compound [22,23,24] but also because it can be administered in various ways (creams, ointments, sprays or oxide dispersions) [25,26,27].

Most of the problems of the known means of administration are related to the unpleasant appearance, irritation of the body surfaces generated by the matrix, rapid loss by friction, reduced persistence time and the need for short-term administration [14,15,23,26]. 

The research in this study was initiated after the loss of membrane material, in the aqueous contact phases, of a liquid membrane system based on n-alkyl alcohols–oxide nanoparticles containing silver and 10–undecenoic acid [28,29,30]. If, in the considered membrane system, the loss of membrane material (silver ions and 10–undecenoic acid) was a disadvantage of using that device, the present research aims at the simultaneous release, either from a polymeric membrane or through an impregnated polymeric membrane, of the two chemical species.

A polymer matrix can ensure the stability of the administration system and mechanical and thermal resistance and can keep the target chemical species in the desired place for a long time.

Thus, this paper studies the simultaneous release of silver ions and 10–undecenoic acid from a membrane system with a matrix of cellulose derivatives (cellulose acetate (CA) and polysulfone (PSf)) with inclusions of oxide nanoparticles containing silver and 10–undecenoic acid as a dispersion medium.

The use of 10–undecenoic acid dispersion with oxide nanoparticles (magnetic) containing silver can ensure both the release of active substances and the possibility of fixing the obtained impregnated membrane to various surfaces with the help of the magnetic field.

## 2. Materials and Methods

### 2.1. Reagents and Materials

#### 2.1.1. Reagents

All reagents and organic compounds used in the presented work were of analytical grade. They were purchased from Merck (Merck KGaA, Darmstadt, Germany): hydrochloric acid, silver nitrate, iron wires, sodium chloride, sodium hydroxide, dimethylformamide (DMF), ethylic alcohol, 10–undecen–1–ol (UDAl), 10–undecenoyl chloride (UDCl) and 10–undecenoic acid (UDAc).

The characteristics of the organic compounds used in the silver ion release study are presented in Table 1.

The purified water characterized by 18.2 μS/cm conductivity was obtained with a RO Millipore system (MilliQ^®^ Direct 8 RO Water Purification System, Merck, Darmstadt, Germany).

#### 2.1.2. Materials

Polymeric materials were polysulfone (PSf), transparent pellets, Mw = 35,000 g/mol, ρ = 1.24 g/cm^3^ (Sigma-Aldrich, St. Louis, MO, USA); cellulose acetate (CA), powder, Mw = 50,000 g/mol, ρ = 1.3 g/cm^3^, (Sigma-Aldrich, St. Louis, MO, USA) (Table 2).

### 2.2. Methods

#### 2.2.1. Obtaining and Characterizing Support Membranes

**Figure 2:**
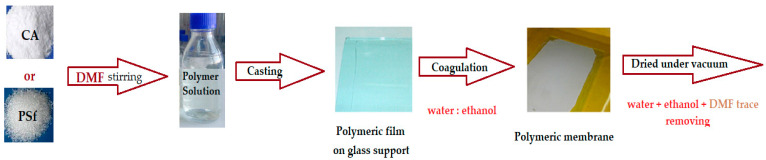
Schematic representation of the procedure for obtaining polymer membranes.

The matrix polymeric membranes were prepared by inversion technique from polymer solution films (10% weight) in dimethylformamide, coagulated in ethanol coagulation bath: water (1v/1v), the phase inversion being previously presented in detail [31,32]. After abundant washing with deionized water and storage for 48 h in pure water, the obtained membranes were dried in a vacuum of 100 mmHg for 72 h. The sequence of operations for obtaining polymeric membranes is shown schematically in Figure 2.

The procedure of obtaining membranes ensures a good surface quality (without wrinkles, surface defects or polymer agglomerations), high porosity and relatively uniform thickness (Table 3).

The vacuum dried membrane does not contain traces of solvent detectable by Fourier transform infrared spectroscopy.

The dry membranes were cut to a size of 100 mm × 100 mm for impregnation with a dispersion of silver-containing oxide nanoparticles.

The general characteristics are presented in Table 3, determined by scanning electron microscopy (SEM), in section and on the surface intended for contact with the expected working environment [33], by measuring the thickness with a micrometer [34] and determining the porosity by gravimetric method [34,35].

#### 2.2.2. Obtaining and Characterizing Oxide Nanoparticles Containing Silver

The iron-based magnetic nanoparticles were obtained by the electrochemical method, previously presented in detail [36,37]. In this case, the electrolysis with iron electrodes was performed in pure water (to obtain magnetic nanoparticles of iron oxides) and in a silver nitrate electrolyte of 10^−3^–10^−1^ mol/L. In the particular case of the present paper, the aim was to obtain oxide nanoparticles with variable silver content.

The nanoparticles obtained by the electrochemical method are dialyzed in a cylindrical Visking membrane (Medicell Membranes Ltd., London, UK) to neutral pH. After magnetic recovery and washing with ethanol, the nanoparticles were dried at room temperature by standing in an oven with laminar airflow. The essential characteristics (morphology, average size and silver content) necessary for use in impregnating the considered membranes are presented in Table 4.

#### 2.2.3. Obtaining the Impregnated Membrane and the Procedure for Evaluating the Release Effect

**Figure 3:**
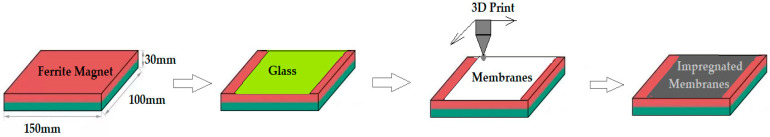
Schematic representation of the procedure for obtaining impregnated membranes.

The dry polymer membranes, having the size of 100 mm × 100 mm, were placed on glass for chromatographic use, and under this was placed a ferrite with high magnetization (150 mm × 100 mm × 30 mm, power of 20 Kg) and were impregnated with a dispersion of iron oxide nanoparticles containing about 0.5–1.5% silver (NP) [36,37], in the desired organic solvent by dosing with a 3D printer programmed for a constant deposition rate (Figure 3).

Three-dimensional printing ensures uniformity of dispersion distribution and avoidance of excess dispersion on the membrane surface. The use of the magnetic field ensures the maintenance of the dispersion in the membrane pores and avoids the loss of nanoparticles during the experiments of controlled release of silver ions and 10–undecenoic acid.

Dispersions of oxide nanoparticles 5 g NP/5 g organic compound from Table 1, impregnated on cellulose acetate and polysulfone membranes, lead to symbolized as in Table 2.

The membranes were cut into 1 cm^2^ disks, containing an average of 0.05 g nanoparticles and 0.05 g organic dispersion compound.

**Figure 4:**
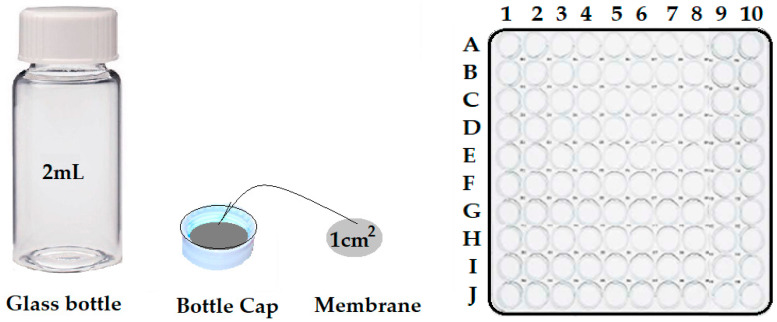
Schematic representation of the membrane arrangement of the lid and the positioning of the sample bottles for the controlled release of silver ions/10–undecenoic acid.

The impregnated membrane discs were placed in the lids of 2 cm^3^ glass bottles. Then, 1.0 mL of controlled pH and ionic strength aqueous solution was introduced into the glass bottles, and the cap was sealed with an impregnated membrane and placed with the cap down in a cup in which 100 bottles could be inserted simultaneously (Figure 4).

The cup of bottles was positioned centrally on the ferrite, also used to impregnate the membranes, to ensure that the nanoparticles were maintained in the support membrane throughout the study, regardless of the amount of dispersion solvent that would be extracted in the test solutions.

Seven bottles were retrieved daily for analysis so that the results of the silver analysis could be mediated, and three bottles were stored as control samples. Analyses using an atomic absorption spectrometer in order to determine silver were performed independently by two researchers, and the devices used were calibrated daily with a standard solution. The validation of the results was performed periodically by electrochemical and/or UV-Vis methods at an independent laboratory.

UV-Vis spectrometric analysis to determine 10–undecenoic acid was performed daily and independently by two researchers, with periodic validation of the results being performed by gas chromatography at an independent laboratory.

### 2.3. Equipment

The surface characteristics of the membranes were determined with scanning electron microscopy (SEM) equipped with a probe for energy dispersive spectroscopy analysis (EDX). Hitachi S4500 system (Hitachi High-Technologies Europe GmbH, Krefeld, Germany) was used [38,39].

The electrochemical processes for silver–iron nanoparticle obtaining were followed up with a PARSTAT 2273 Potentiostat (Princeton Applied Research, AMETEK Inc., Berwyn, PA, USA). A setup based on a glass cell with three electrodes was used [36,37].

Determination and monitoring of pH for every stock solution were achieved using a conductance cell or combined selective electrode (HI 4107, Hanna Instruments Ltd., Leighton Buzzard, UK) and a multi-parameter system (HI 5522, Hanna Instruments Ltd., Leighton Buzzard, UK) [36].

To assess and validate the content in metal ions, an atomic absorption spectrometer AAnalyst 400 AA Spectrometer (Perkin Elmer Inc., Waltham, MA, USA) with WinLab32–AA software (Perkin Elmer Inc., Waltham, MA, USA), with a single-element hollow-cathode lamp was used. The operating current was set up at 2 mA, wavelength 248.3 nm and 0.2 nm spectral bandwidth for determining the iron content. For silver, the experimental parameters were 328.1 nm wavelength and 0.7 nm spectral bandwidth at an operating current of 5 mA [37,40,41,42].

The UV-Vis spectra of the 10–undecenoic acid samples were recorded for a wavelength ranging from 200 to 800 nm, at room temperature, using 10 mm quartz cells on CamSpec M550 spectrometer (Spectronic CamSpec Ltd., Leeds, UK) [43].

Additionally, the UV–Vis validation analysis of the 10–undecenoic acid solutions was performed on a dual-beam UV equipment–Varian Cary 50 (Agilent Technologies Inc., Santa Clara, CA, USA) at a resolution of 1 nm, spectral bandwidth of 1.5 nm and a scan rate of 300 nm/s [36,37].

All determinations were performed on the same day, for each scheduled experiment, by two experienced analysts from different laboratories, based on 7 specimens taken for each sample, and to ensure the quality of chemical measurements, the specific EURACHEM guide was followed [44,45].

## 3. Results and Discussions

Delayed and/or controlled release of chemical species of pharmaceutical interest is a particular aspect of membrane use. For the system from which a compound is released through the membrane has a single target component, both the membrane transport experiments and their modeling have been extensively and thoroughly studied [46,47,48,49,50]. For systems that simultaneously release two or more target chemical species, both experiments, but especially process modeling, become much more complex [51,52,53].

**Figure 5:**
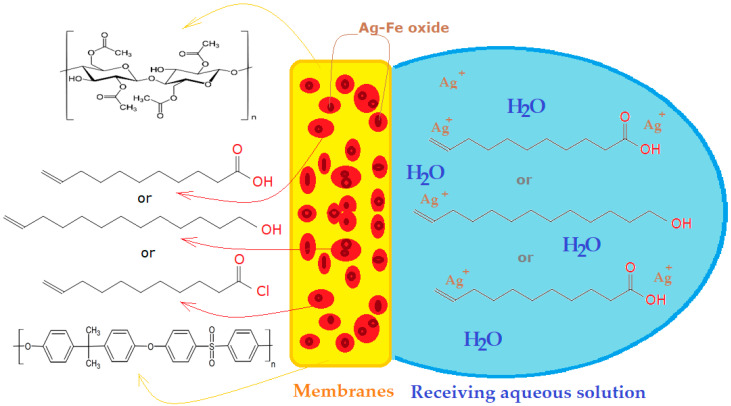
Schematic representation of the system of releasing silver ions and 10–undecenoic acid from the impregnated polymer membrane in an aqueous solution.

In the case studied in this paper, the aim was to release silver ions and 10–undecenoic acid from a membrane matrix based on cellulose acetate or polysulfone. The materials were chosen for the watering difference: the polysulfone being hydrophobic and the cellulose acetate being hydrophilic. Silver ions and 10–undecenoic acid were introduced into the membrane polymer matrix as a dispersion of iron and silver oxide nanoparticles in 10–undecenoic acid. The receiving solution of the two chemical species was aqueous in nature, with predetermined pH and sodium chloride concentration (Figure 5).

The experiments monitored the effect of the nature of the organic compound and the membrane polymer matrix, the influence of pH and ionic strength of the receiving solution and the contribution of silver concentration in oxide nanoparticles to the concentration of target substances released in a considered aqueous system.

### 3.1. The Influence of the Organic Compound and the Polymeric Matrix on the Release of Silver Ions

The organic compounds: 10–undecen–1–ol (UDAl), 10–undecenoyl chloride (UDCl), and 10–undecenoic acid (UDAc), in which the dispersion of oxide nanoparticles containing 1.63% silver was performed, were chosen so as to highlight the effect of the functional groups of 10–undecenoic acid (alkene and carboxylic) on the release of silver ions in aqueous solutions can be highlighted.

The aqueous receiving solutions had the required pH values: 5.0, 6.8, 7.0 and 7.2, specific to determinations for systems in contact with biological environments [54,55].

**Figure 6:**
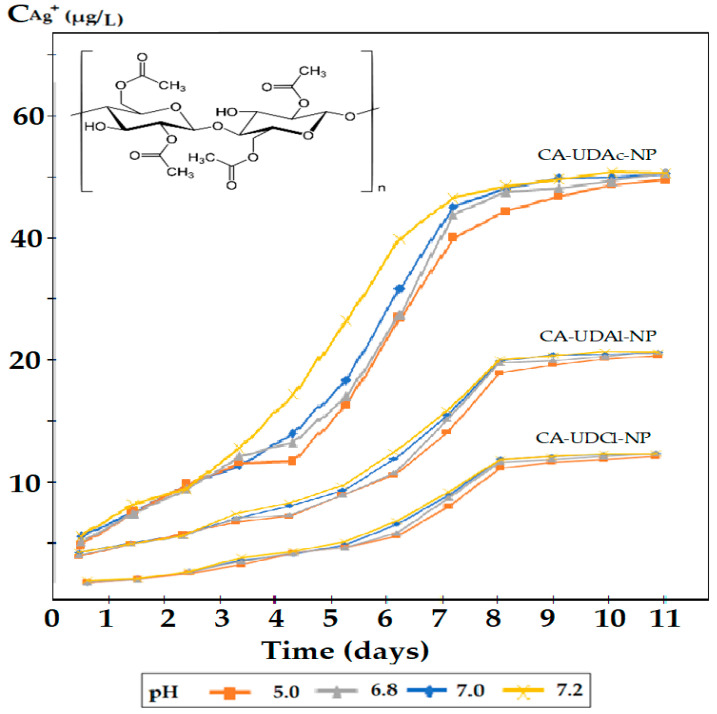
Concentration of silver ions released in the receiving aqueous phase of pH: 5.0, 6.8, 7.0 and 7.2 for cellulose acetate membrane in the case of 10–undecenoic acid (UDAc), 10–undecen–1–ol (UDAl) or 10–undecenoic chloride (UDACl) as dispersants and oxide nanoparticles with 1.63% silver.

**Figure 7:**
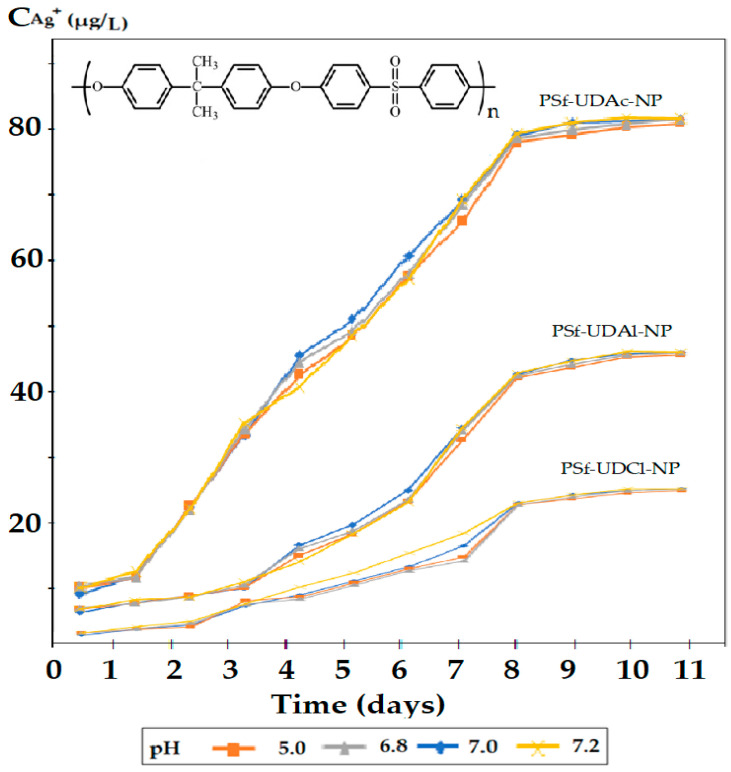
Concentration of silver ions released in the receiving aqueous phase of pH: 5.0, 6.8, 7.0 and 7.2 for polysulfone membrane in the case of 10–undecenoic acid (UDAc), 10–undecen–1–ol (UDAl) or 10–undecenoic chloride (UDACl) as dispersants and oxide nanoparticles with 1.63% silver.

The obtained results were represented for all nanoparticle dispersing agents, for the cellulose acetate matrix (Figure 6) and the polysulphone matrix (Figure 7).

The amount of silver ions released into the environment depends on both the polymeric matrix and the three dispersants used (Figure 6 and Figure 7). The cellulose acetate matrix (Figure 6) released the silver ions harder than the polysulphone matrix (Figure 7), most likely due to the interaction of silver ions with the acetyl and hydroxyl groups in cellulose acetate.

On the other hand, the three dispersants had an important contribution to the controlled release, which can be explained by their solubility in the aqueous medium and the contact angle (hydrophobicity) it generates for impregnated membranes (Table 2).

The silver ion concentration plateau in the receiving aqueous solution, observed in all experiments, after about 8 days of contact of the membranes impregnated with the aqueous medium (Figure 6, Figure 7 and Figures S1–S3), had three causes: achieving a balance of Donnan type between the concentration (activity) of ions in the membrane and the aqueous receptor solution, depletion of silver ions from the surface of nanoparticles in the immediate vicinity of the interface with the receptor solution and/or achieving complex interphase balance.

In the Supplementary Materials, the results obtained for the two types of membrane polymeric matrices (cellulose acetate and polysulfone) and the three organic compounds (Figures S1–S3) show the evolution of the release of silver ions in the considered aqueous systems. In all dispersant cases, the cellulose acetate membrane compounds (Figures S1–S3) release silver ions harder than the polysulfone membrane compounds (Figures S1–S3).

The lower release of silver ions in the case of cellulose acetate can be correlated with the more pronounced interaction of silver ions on the hydrophilic support matrix, which retains them both by ion–dipole interactions (silver-carbonyl or hydroxyl groups) and by hydrogen bonds between the hydroxyl and/or carbonyl groups and the hydration coating of the aqua-complex silver ion. For both matrices, the concentration of silver ions after the third day of exposure is sufficient to ensure both the biocidal (bactericidal—0.1 µg/L) and the cytotoxic effect (1.60 µg/L). Depending on the application pursued and the environment in which both polymer matrices will be used, they may be useful, but in the present study, the experiments will continue only with the polysulfone matrix.

From the point of view of the 10–undecenoic acid dispersant, it allows the release of silver ions in the aqueous phase much more easily (Figure 5) compared to 10–undecen–1–ol (Figure 6) and of almost an order of magnitude more than 10–undecenoyl chloride (Figure 7), when comparing polysulfone (Figure 5) with cellulose acetate (Figure 7).

The results of the release of silver ions depending on the nature of the dispersant correlate with the solubility of the dispersants in water (Table 1), but also with the possibility of interaction of silver ions with these organic compounds. They were chosen because they can interact with the silver ion through both the alkenic group and the carboxyl or hydroxyl groups. Basically, the sequence observed for the release of silver ions (see Figure 5, Figure 6 and Figure 7): 10–undecenoic acid (UDAc) > 10–undecen–1–ol (UDAl) >>> 10–undecenoyl chloride (UDCl) shows us that the first compound has a strong interaction center (carboxyl group) as well as a medium interaction center (alkenic group), the second has two medium interaction groups (hydroxyl and alkylene groups), and the third a group of medium interaction (alkylene) and one low interaction (carbonyl). It is interesting that 10-undecenol chloride, being insoluble in water, does not favor the transfer of silver ions in the aqueous receiving solution. 

Figure 5, Figure 6 and Figure 7 do not clearly show the effect of the pH of the receiving aqueous phase on the release of silver ions, most likely due to the rather narrow range of the chosen value. However, it can be seen that at pH = 5.0, the release of silver ions is slightly faster. The capping of the concentration in the receiving aqueous phase after the seventh working day indicates the achievement of a trans-membrane equilibrium that depends on both the type of polymeric matrix and the organic dispersing compound.

The simultaneous release of silver ions and 10–undecenoic acid from the impregnated membrane is a complex process characterized by several equilibria in which pH plays an important role (1)–(5):

(H_2_C = CH-(CH_2_)_8_-COOH) _M_ + (HOH) _RP_ ⇌ (H_2_C = CH-(CH_2_)_8_-COO^−^) _RP_ + (H_3_O^+^) _RP_ (1)

(H_2_C = CH-(CH_2_)_8_-COOH) _M_ + (HO^–^) _RP_ ⇌ ((H_2_C = CH-(CH_2_)_8_-COO^−^) _RP_ + (HOH) _RP_ (2)

(H_2_C = CH-(CH_2_)_8_-COOH) _M_ + (Ag^+^) _NP_ + (HOH) _RP_ ⇌ (H_2_C = CH-(CH_2_)_8_-COO^–^Ag^+^) _RP_ + (H_3_O^+^) _RP_ (3)

(H_2_C = CH-(CH_2_)_8_-COO^–^) _M_ + 2(Ag^+^) _NP_ ⇌ (Ag^+^H_2_C = CH-(CH_2_)_8_-COO^–^Ag^+^) _RP_ (4)

(Ag^+^) _NP_ + (Cl) _RP_ ⇌ (AgCl) _NP_ (5)

(Ag^+^) _NP_ + 2(Cl^–^) _RP_ + (HOH) _M_ ⇌ ([AgCl_2_]^–^) _RP_ + (HOH) _RP_ (6)

M denotes the membranes, NP the oxide nanoparticles and RP the receiving phase.

Other interphase equilibria can certainly be considered, but those already presented justify the obtained results and are illustrated in Figure 5, Figure 6 and Figure 7 and Figure 9. The defining factors are two centers of the interaction of 10–undecenoic acid (4), but also the possibility of silver ions to be complexed in excess by chloride ions (6).

### 3.2. Influence of Membrane Support Morphology and Silver Content of Oxide Nanoparticles on the Release of Silver Ions in Aqueous Solution

**Figure 8:**
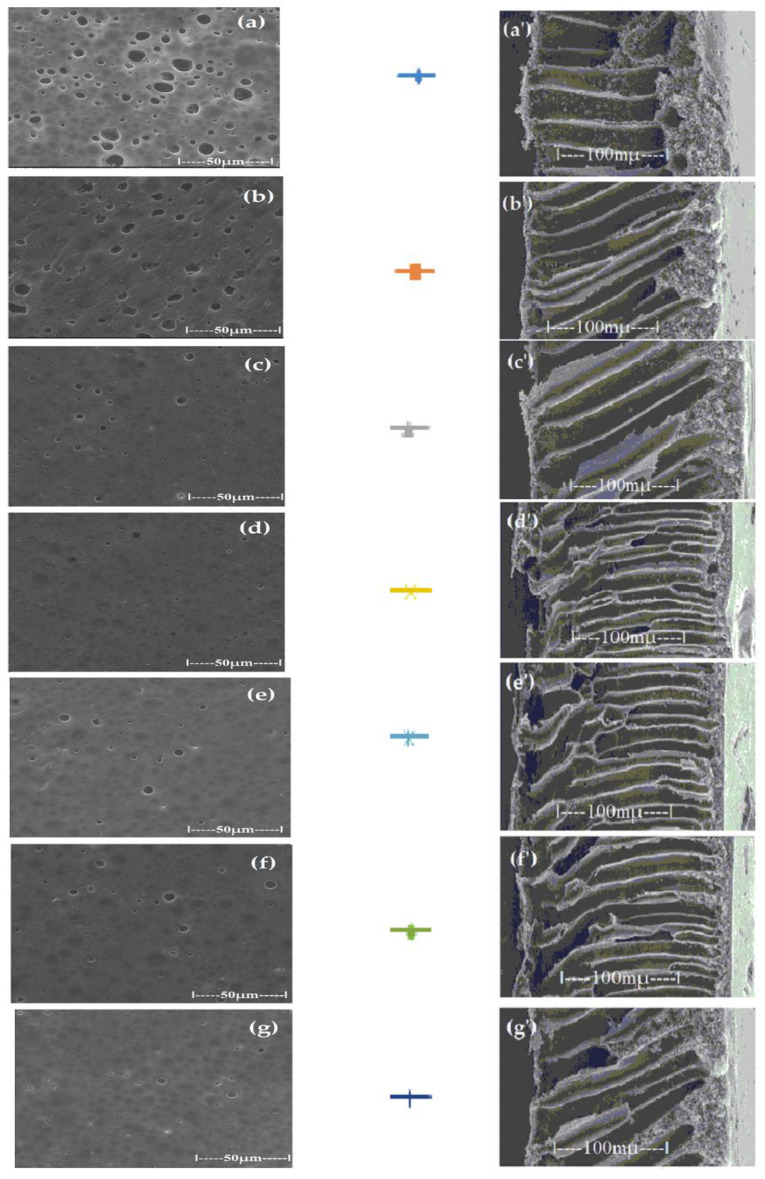
Morphology of polysulfone support membranes obtained by different exposure to the polymeric film before coagulation: (**a**–**g**) bottom views and (**a’**–**g’**) cross-sections.

**Figure 9:**
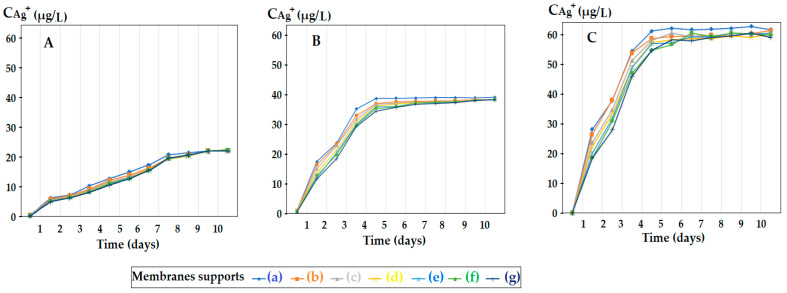
Concentration of silver ions released in the receiving aqueous phase of pH = 7, as a function of time, for seven morphologies of the polysulfone support and for oxide nanoparticles containing silver: (**A**) 0.55%, (**B**) 1.12% and (**C**) 1.63%.

For the polysulfone support membrane matrix, which provided the highest concentrations of released silver ions, the effect of macro-porous surface morphology (Figure 8 and Figure S4) on the release process using the three types of oxide nanoparticles was studied, containing 0.55%, 1.12% and 1.63% silver, respectively (Figure 9).

The morphology of support membranes, which is obtained by controlling by adjusting the standing time of the film in the medium before coagulation [56,57], moderately influences the release in the aqueous test solution during 2–5 days of monitoring. The concentration level of silver ions is determined by their concentration in oxide nanoparticles in the 10–undecenoic acid dispersion. Thus, the higher the concentration of silver in the oxide nanoparticles, the higher the limit of the concentration of silver ions in the aqueous solution of pH = 7 (Figure 9A–C).

These observations are in agreement with the previous results [28,29,30] and are correlated with the size and distribution of macro-porous pores of the membrane surface on which the dispersion of silver-containing oxide nanoparticles is impregnated in the first days of contact (Figure 8). At a longer operating time, a balance is achieved between the concentration of ions in the impregnated membrane and that of the ions in the receiving aqueous system.

It is interesting that for the case study, the ratio of the concentration of silver ions in the receiving solution and, respectively, in the concentration of silver in the oxide nanoparticles is relatively constant, suggesting a Donnan-type transmembrane equilibrium [58,59,60].

The data provided by these experiments allow the control of the limiting concentration of silver ions in a given aqueous solution by adjusting the concentration of silver in the oxide nanoparticles.

For the practical use of these experimental observations, the following must be taken into account: the nature of the polymer from which the support membrane is made, the type of the organic compound in which the oxide nanoparticles are dispersed and the concentration of silver in these nanoparticles.

### 3.3. The Influence of the Receiving Phase pH of the Silver Ions and 10–Undecenoic Acid Simultaneous Release

**Figure 10:**
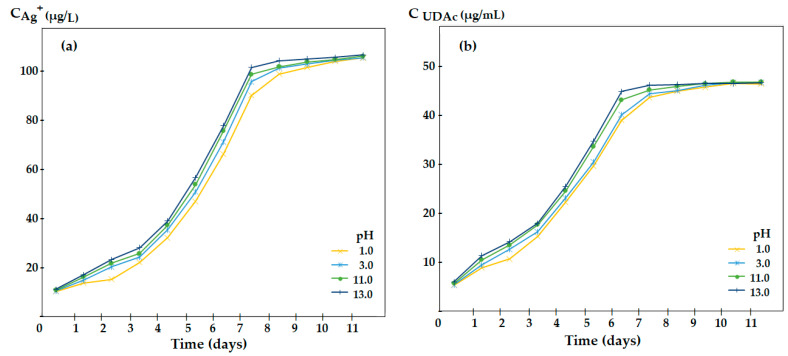
Concentration of silver ions (**a**) and of 10–undecenoic acid (**b**) released in the receiving aqueous phase of variable pH, depending on the type of contact.

For the polysulfone–10–undecenoic acid–aqueous solution matrix system, the influence of extreme pH on the release of silver ions and of 10–undecenoic acid from a membrane impregnated with oxide nanoparticles containing 1.63% silver and 10–undecenoic acid as a dispersing agent was followed (Figure 10).

pH values such as 1, 3, 11 and 13 are all allowed by the polysulfone membrane support, which is resistant over the entire pH range [61,62].

The test of the polysulfone–10–undecenoic acid–oxide nanoparticles system containing 1.63% silver for the simultaneous controlled release of silver ions and 10–undecenoic acid shows that at extreme pH values, the concentration of silver ions covering the bactericidal and cytotoxic requirements is obtained quickly, and the concentration level after 7 days of contact is slightly higher than at a pH close to neutral pH (Figure 5 and Figure 10a). At the studied pH values, the release of 10–undecenoic acid follows the shape of the curves for silver ions, but its concentration is lower (Figure 10b). However, the 10–undecenoic concentration slowly exceeds the limit of its solubility in pure water, thus contributing to the fungicidal effect of the studied system [63]. The reason for which the compounds derived from 10–undecenoic acid allow the release of smaller amounts of silver ions (equilibria (7) and (8)) can be understood in this way: they have a single center of interaction organic compound–silver ions, compared to 10–undecenoic acid which may have two such centers of interaction (equilibria (3) and (4)):

(H_2_C = CH-(CH_2_)_8_-COCl) _M_ + 2(Ag^+^) _NP_ ⇌ (Ag^+^H_2_C = CH-(CH_2_)_8_-COCl) _RP_ (7)

(H_2_C = CH-(CH_2_)_8_-CHOH) _M_ + 2(Ag^+^) _NP_ ⇌ (Ag^+^H_2_C = CH-(CH_2_)_8_-CH_2_OH) _RP_ (8)

### 3.4. The Influence of the Ionic Strength of the Silver Ions and 10–Undecenoic Acid Simultaneous Release

**Figure 11:**
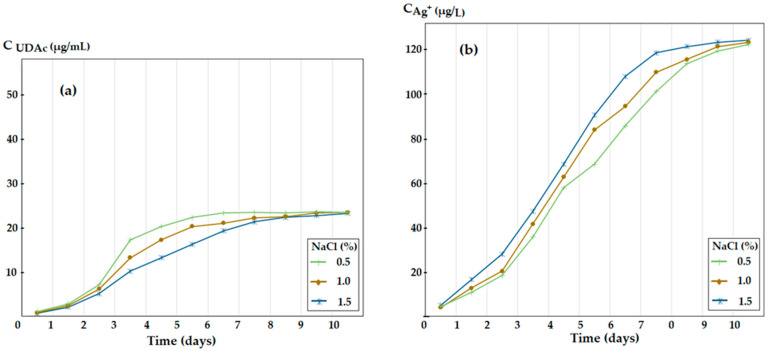
Concentration of 10–undecenoic acid (**a**) and of silver ions (**b**) released in the receiving aqueous phase, of variable ionic strength (NaCl), depending on the type of contact.

Equilibria (5), but especially (6), required the study of the influence of sodium chloride concentration on the receiving aqueous phase. The use of sodium chloride is justified both by the fact that no additional anions are introduced compared to the study in the previous section and by the similarity with the biological environment in which this impregnated membrane could come into contact. For a matrix system of polysulfone–10–undecanoic–aqueous solution, the influence of electrolyte concentration (NaCl) on the release of silver ions and 10–undecenoic acid from the membrane impregnated with oxide nanoparticles containing 1.63% silver in the dispersing agent was followed (Figure 11). In order not to alter the results of the study, the chloride ion that could come from the receiving solution acidified with hydrochloric acid, the experiments were performed with a receiving phase of concentrations of 0.5, 1.0 and 1.5% (gravimetric) sodium chloride in pure water. Both the concentration of 10–undecenoic acid (Figure 11a) and that of the silver ions (Figure 11b) released in the receiving solution were monitored.

The presence of sodium chloride in the receiving aqueous phase negatively influences the release of 10–undecenoic acid (Figure 11a), the limiting concentration at 10 days (approx. 20 µg/mL) being much lower than that obtained in non-saline solution for the same tracking period (approx. 45 µg/mL). This observation is in agreement with previous data, which showed that the loss of 10–undecenoic acid in a membrane can be reduced by using electrolyte additives (NaCl or NaNO3) [28,29,30].

On the other hand, the effect of sodium chloride is favorable for the release of silver ions in the receiving aqueous phase (Figure 11b), the concentration limit at more than 10 days of contact between the impregnated membrane and the aqueous saline solution exceeding 120 µg/L. What is noteworthy in this case is the much faster release of silver ions in solution (Figure 11b) compared to Figure 5, Figure 6 and Figure 7 and Figure 10a. Consequently, the equilibrium (6), forming the complex [AgCl2]^–^, is completely shifted to the left due to excess of chloride ions.

The two opposite aspects of the release of silver ions and 10–undecenoic acid from the membrane impregnated with oxide nanoparticles containing silver in the dispersing agent must be considered for use in saline biological media, as the bactericidal effect improves, and the fungicidal effect decreases. For example, when using the system for high-performance athletes who exert salts in perspiration, the fungicidal effect of 10–undecenoic acid is diminished.

The application of the system based on the controlled release of silver ions and 10–undecenoic acid from membranes impregnated with oxide nanoparticles containing silver in the dispersing agent requires extensive studies on release in real biological mediums: blood, saliva, perspiration or urine.

For instance, in order to answer to the necessities of sports medicine, the tests with various biological fluids must include other polymeric matrices (polylactic acid, polyvinyl alcohol, polyvinyl acetate, polyamide) or dispersion media than those addressed in the present paper.

## 4. Conclusions

This paper studies the effect of the polymer matrix on the simultaneous release of silver ions and 10–undecenoic acid in an aqueous medium of controlled pH and ionic strength.

Polymeric matrices consisting of cellulose acetate (CA) and polysulfone (PSf) were impregnated with oxide magnetic nanoparticles containing silver ions and 10–undecenoic acid. The polymeric matrices were prepared as microporous membranes by the phase inversion method, and the oxide nanoparticles were obtained by electrolysis of silver nitrate solutions with iron electrodes.

Impregnated membranes were made by printing the dispersion of oxide magnetic nanoparticles containing 0.63, 1.12 and 1.63% (mass) silver in organic compounds: 10–undecen–1–ol (UDAl), 10–undecenoyl chloride (UDCl) and 10–undecenoic acid (UDAc).

The study of the release of silver ions in aqueous solutions of imposed pH and sodium chloride concentration carried out over a maximum of 11 days shows that after the first day, the silver ions reach the concentration that ensures the bactericidal and/or cytotoxic effect, and after 5–7 days, the concentration is capped at values that are determined by the matrix type and morphology, the silver concentration of the nanoparticles and the nature of the organic compound. Higher silver concentration limit values are obtained for the polysulfone matrix, 10–undecenoic acid dispersant, neutral or basic pH, or higher sodium chloride concentration.

For the polysulfone–10–undecenoic acid–aqueous solution matrix, we monitored the influence of the electrolyte concentration (NaCl) on the simultaneous release of silver ions and 10–undecenoic acid from the membrane impregnated with oxide nanoparticles containing 1.63% silver in the dispersing agent. 

The presence of sodium chloride in the aqueous receiving phase negatively influences the release of 10–undecenoic acid, but on the other hand, the effect of sodium chloride is favorable for the release of silver ions in the receiving aqueous phase. The two antagonistic aspects of the release of silver ions and 10–undecenoic acid from the membrane impregnated with oxide nanoparticles containing silver in the dispersing agent must be considered for use in saline biological media as the bactericidal effect improves and the fungicidal effect decreases.

The application of the controlled release system of silver ions and 10–undecenoic acid from polymeric membranes impregnated with silver-containing oxide nanoparticles in the dispersing agent requires extensive studies on the release in real biological environments: blood, saliva, perspiration or urine.

## Figures and Tables

**Table 1:** The characteristics of the used organic compounds.

Organic Compounds	Name and Symbol	Molar Mass (g/mol)	Density (g/mL)	Solubility in Water (g/L)	pKa
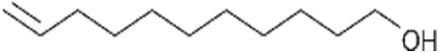	10–undecen–1–ol (UDAl)	170.29	0.848	0.014 estimated 0.044 exp.	16.84
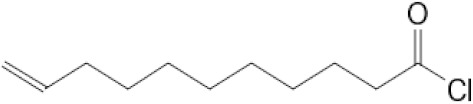	10–undecenoyl chloride (UDCl)	202.72	0.944	–	none
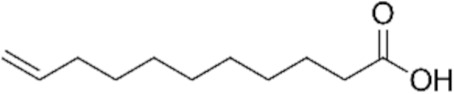	10–undecenoic acid (UDAc)	184.28	0.912	0.019 estimated 0.0737 exp.	5.02

**Table 2:** The characteristics of polymers for the obtained membranes.

Polymer	Chemical Formula	Molar Weight (Da)	Membrane Symbols	Contact Angle *) (θ°)
Cellulose acetate (CA)	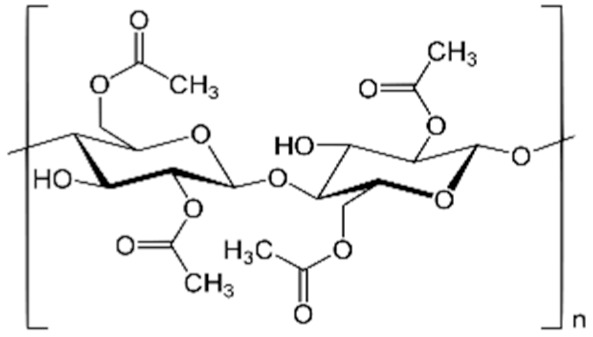	50,000	CA-UDAc-NP CA-UDAl-NP CA-UDCl-NP	43 ± 2 52 ± 2 60 ± 2
Polysulfone (PSf)	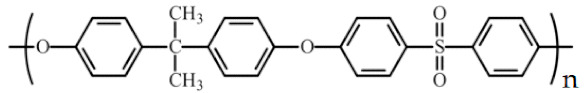	35,000	PSf-UDAc-NP PSf-UDAl-NP PSf-UDCl-NP	65 ± 2 71 ± 2 76 ± 2

*) Contact angle measurements for the considered membranes (with distilled water).

**Table 3:** The characteristics of the membrane support.

Membrane	Scanning Electron Microscopy (SEM)		Thickness (µm)	Porosity (%)
	Cross-Section	Bottom		
Cellulose acetate (CA)	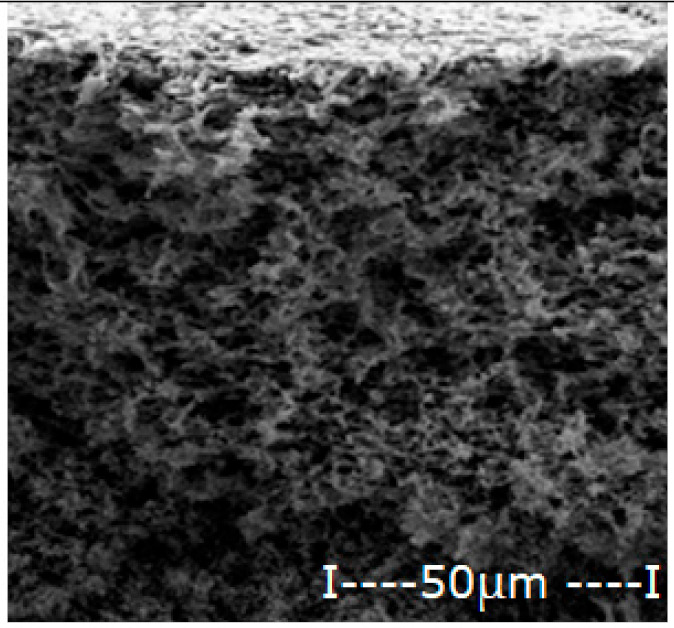	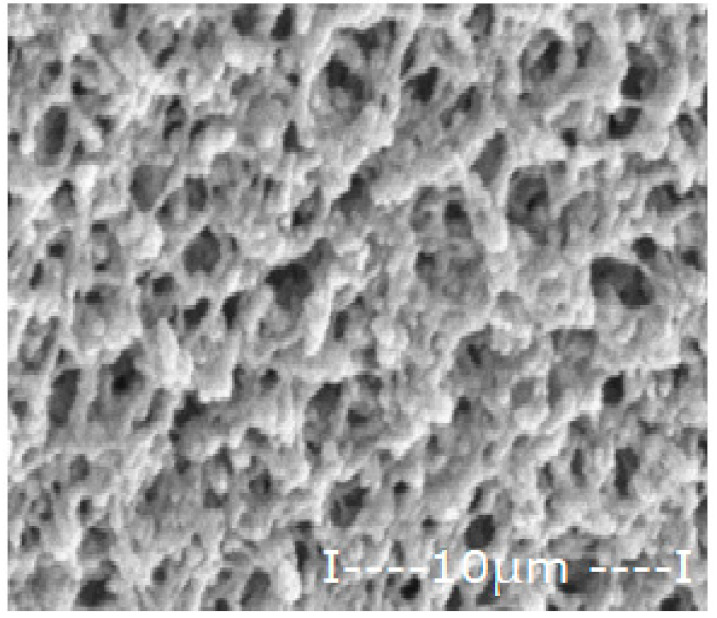	162 ± 5	82 ± 3
Polysulfone (PSf)	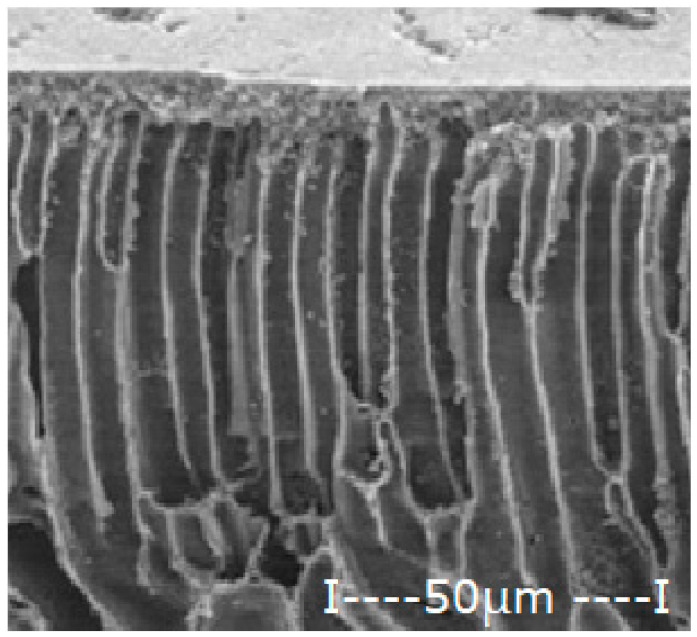	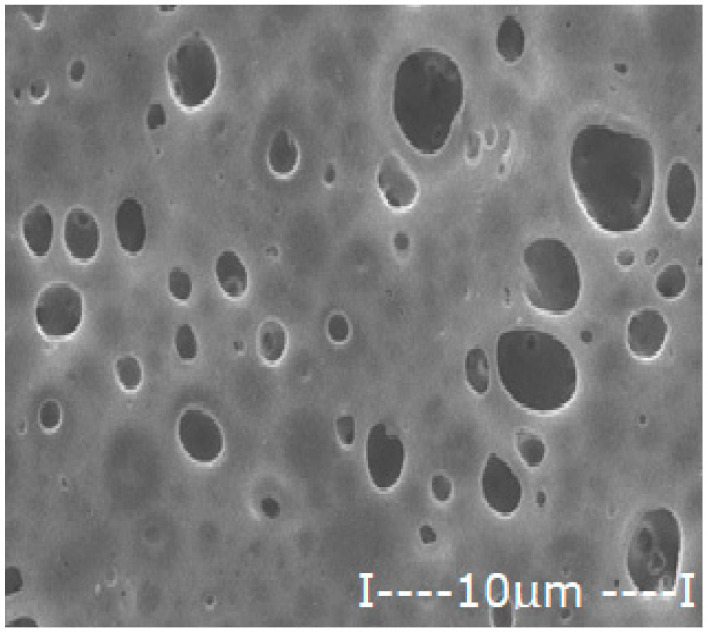	165 ± 3	76 ± 2

**Table 4:** The characteristics of the silver–iron oxide nanoparticles.

Oxide Nanoparticle (Ag–NP)	Scanning Electron Microscopy (SEM)	Medium Diameter (nm)	Medium Silver Content (%)
NP0.55	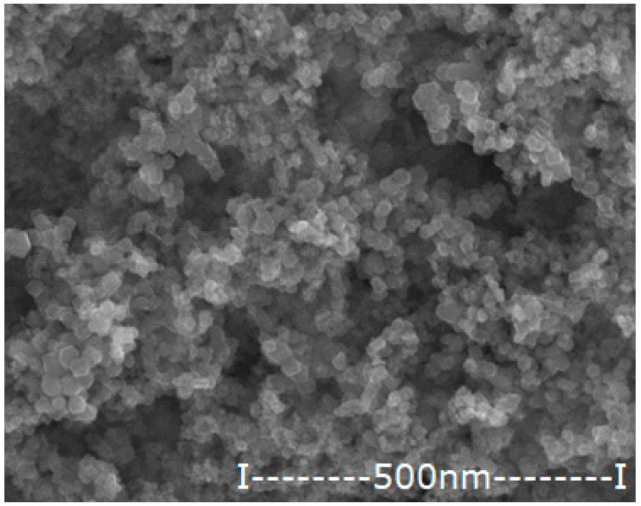	42.8	0.55
NP1.12	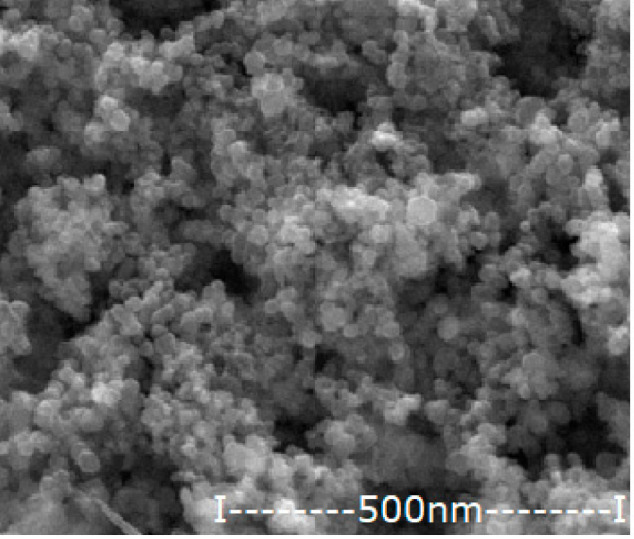	45.4	1.12
NP1.63	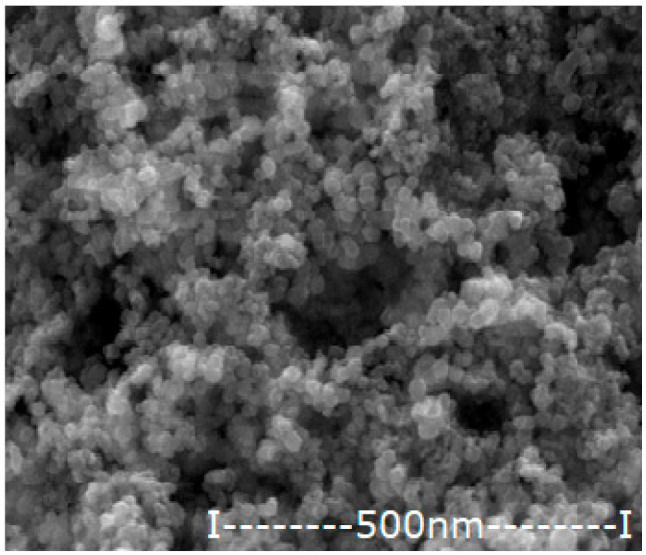	47.1	1.63

## Data Availability

Not applicable.

## Supplementary Materials

The following supporting information can be downloaded at: https://www.mdpi.com/article/10.3390/membranes12060557/s1, **Figure S1**: Concentration of silver ions released in the receiving aqueous phase of pH: 5.0; 6.8; 7.0; and 7.2 for cellulose acetate and polysulfone membrane in the case of 10–undecenoic acid as dispersant and oxide nanoparticles with 1.63% silver); **Figure S2**: Concentration of silver ions released in the receiving aqueous phase of pH: 5.0; 6.8; 7.0; and 7.2 for cellulose acetate and polysulfone membrane in the case of 10–undecen–1–ol as dispersant and oxide nanoparticles with 1.63% silver; **Figure S3**: Concentration of silver ions released in the receiving aqueous phase of pH: 5.0; 6.8; 7.0; and 7.2 for cellulose acetate and polysulfone membrane in the case of 10–undecenoic chloride as dispersant and oxide nanoparticles with 1.63% silver; and **Figure S4**: Morphology of polysulfone support membranes obtained by different exposure to the polymeric film before coagulation.
