# Supplementary material for: Simultaneous Release of Silver Ions and 10–Undecenoic Acid from Silver Iron–Oxide Nanoparticles Impregnated Membranes
Source: Membranes (Basel). 2022 May 27;12(6):557. doi: 10.3390/membranes12060557 (PMC9227798; doi:10.3390/membranes12060557)
Supplement: Supplementary file 1 [file membranes-12-00557-s001.zip › membranes-1692003-supplementary.pdf]

membranes

Supplementary materials

Simultaneously release of silver ions and 10-undecenoic acid from silver iron – oxide nanoparticles impregnated membranes

Gheorghe Nechifor [1], Alexandra Raluca Grosu [1], Andreea Ferencz (Dinu) [1], Szidonia-Katalin Tanczos [2], Alexandru Goran [1], Vlad-Alexandru Grosu [3,*], Simona Gabriela Bungău [4], Florentina Mihaela Păncescu [1], Paul Constantin Albu [5,*], Aurelia Cristina Nechifor [1]

[1] Analytical Chemistry and Environmental Engineering Department, University Politehnica of Bucharest, Bucharest 011061, Romania, ghnechifor@gmail.com; andra.grosu@upb.ro; andra_d24@yahoo.com; alexandru@santego.ro; florynicorici@yahoo.com; aureliacristinanechifor@gmail.com; (G.N.); (A.R.G.); (A.F.); (A.G.); (F.M.P.); (A.C.N.);

[2] Department of Bioengineering, University Sapientia of Miercurea-Ciuc, Miercurea-Ciuc 500104, Romania, tczszidonia@yahoo.com (S.-K.T.)

[3] Department of Electronic Technology and Reliability, Faculty of Electronics, Telecommunications and Information Technology, University Politehnica of Bucharest 061071, Romania, vlad.grosu@upb.ro (V.-A.G.)

[4] Department of Pharmacy, Faculty of Medicine and Pharmacy, University of Oradea, 410028 Oradea, Romania, sbungau@uoradea.ro (S.G.B.)

[5] Radioisotopes and Radiation Metrology Department (DRMR), IFIN Horia Hulubei, Măgurele 023465, Romania, paulalbu@gmail.com (P.C.A.)

* Correspondence: vlad.grosu@upb.ro; paulalbu@gmail.com;

Citation: Nechifor, G.; Grosu, A.R.; Ferencz , A.; Tanczos, S.-K.; Goran, A.; Grosu, V.-A.; Bungău, S.G.; Păncescu, F.M.; Albu, P.C.; Nechifor, A.C. Simultaneously release of silver ions and 10-undecenoic acid from silver iron – oxide nanoparticles impregnated membranes. *Membranes* **2022**, *12*, 557. https://doi.org/10.3390/membranes12060557

Academic Editor: Pei Sean Goh

Received: 5 April 2022
Accepted: 23 May 2022
Published: 27 May 2022

Publisher's Note: MDPI stays neutral with regard to jurisdictional claims in published maps and institutional affiliations.

Copyright: © 2022 by the authors. Licensee MDPI, Basel, Switzerland. This article is an open access article distributed under the terms and conditions of the Creative Commons Attribution (CC BY) license (https://creativecommons.org/licenses/by/4.0/).

Abstract: The bio-medical benefits of silver ions and 10–undecenoic acid in various chemical-pharmaceutical preparations are indisputable, thus justifying the numerous researches of delayed and / or controlled release. This paper presents the effect of the polymer matrix in the simultaneous release of silver ions and 10–undecenoic acid in an aqueous medium of controlled pH and ionic strength. The study took into consideration polymeric matrices consisting of cellulose acetate (CA) and polysulfone (PSf), which were impregnated with oxide nanoparticles containing silver and 10–undecenoic acid. The studied oxide nanoparticles are nanoparticles of iron and silver oxides obtained by an accessible electrochemical method. The obtained results show that silver can be released, simultaneously with 10–undecenoic acid, from an impregnated polymeric membrane, at concentrations that ensure the biocidal and, respectively, the fungicidal capacity. Concentrations of active substances can be controlled by choosing the polymer matrix or, in some cases, by changing the pH of the target medium. In the studied case, higher concentrations of silver ions are released from the polysulfone matrix, while higher concentrations of 10–undecenoic acid are released from the cellulose acetate matrix. The results of the study show that a correlation can be established between the two released target substances, which is dependent on the solubility of the organic compound in the aqueous medium and the interaction of this compound with the silver ions. The ability of 10–undecenoic acid to interact with the silver ion, both through the carboxyl and alkene groups contributes to the increase in content of the silver ions transported in the aqueous medium.

Keywords: control release; composite membranes; impregnated membranes; silver-iron oxide nanoparticles; silver ions; 10–undecenoic acid; cellulose derivatives; cellulose acetate, polysulfone.

1. *The influence of the organic compound and the polymeric matrix on the release of silver ions*

The organic compounds: 10–undecen–1–ol (UDAl), 10–undecenoyl chloride (UDCl), and 10–undecenoic acid (UDAc) in which the dispersion of oxide nanoparticles containing 1.63 % silver is performed were chosen so as to highlight the effect of the functional groups of 10–undecenoic acid (alkene and carboxylic) on the release of silver ions in aqueous solutions can be highlighted.

The results obtained for the two types of membrane polymeric matrices (cellulose acetate and polysulfone) and the three organic compounds (Figures S1, S2 and S3) show the evolution of the release of silver ions in the considered aqueous systems. In all dispersant cases, the cellulose acetate membrane (Figures S1, S2 and S3) releases silver ions harder than the polysulfone membrane (Figures S1, S2 and S3).

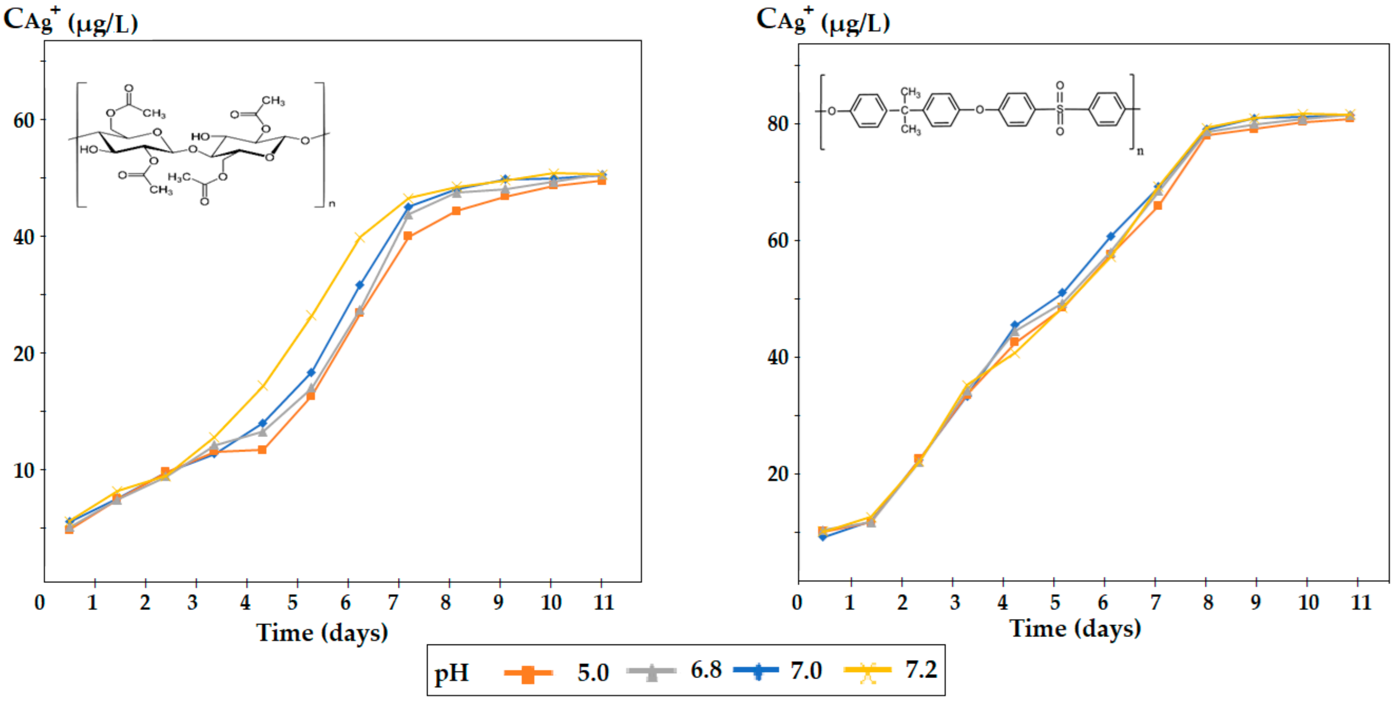

Figure S1. Concentration of silver ions released in the receiving aqueous phase of pH: 5.0; 6.8; 7.0; and 7.2 for cellulose acetate and polysulfone membrane in the case of 10–undecenoic acid as dispersant and oxide nanoparticles with 1.63% silver.

From the point of view of the 10–undecenoic acid dispersant, it allows the release of silver ions in the aqueous phase much more easily (Figures S1), compared to 10–undecen–1–ol (Figure S2) and of almost an order of magnitude more than 10–undecenoyl chloride (Figure 7), when comparing polysulfone (Figure S1) with cellulose acetate (Figure S3).

The results of the release of silver ions depending on the nature of the dispersant correlate with the solubility of the dispersants in water (Table 1), but also with the possibility of interaction of silver ions with these organic compounds. Their choice was made because they can interact with the silver ion through both the alkenic group and the carboxyl or hydroxyl groups. Basically, the sequence observed for the release of silver ions (see Figures 5, 6 and 7): 10–undecenoic acid (UDAc) > 10–undecen–1–ol (UDAl) >>> 10–undecenoyl chloride (UDCl), shows us that the first compound has a strong interaction center (carboxyl group) as well as a medium interaction center (alkenic group), the second has two medium interaction groups (hydroxyl and alkylene groups), and the third a group of medium interaction (alkylene) and one low interaction (carbonyl).

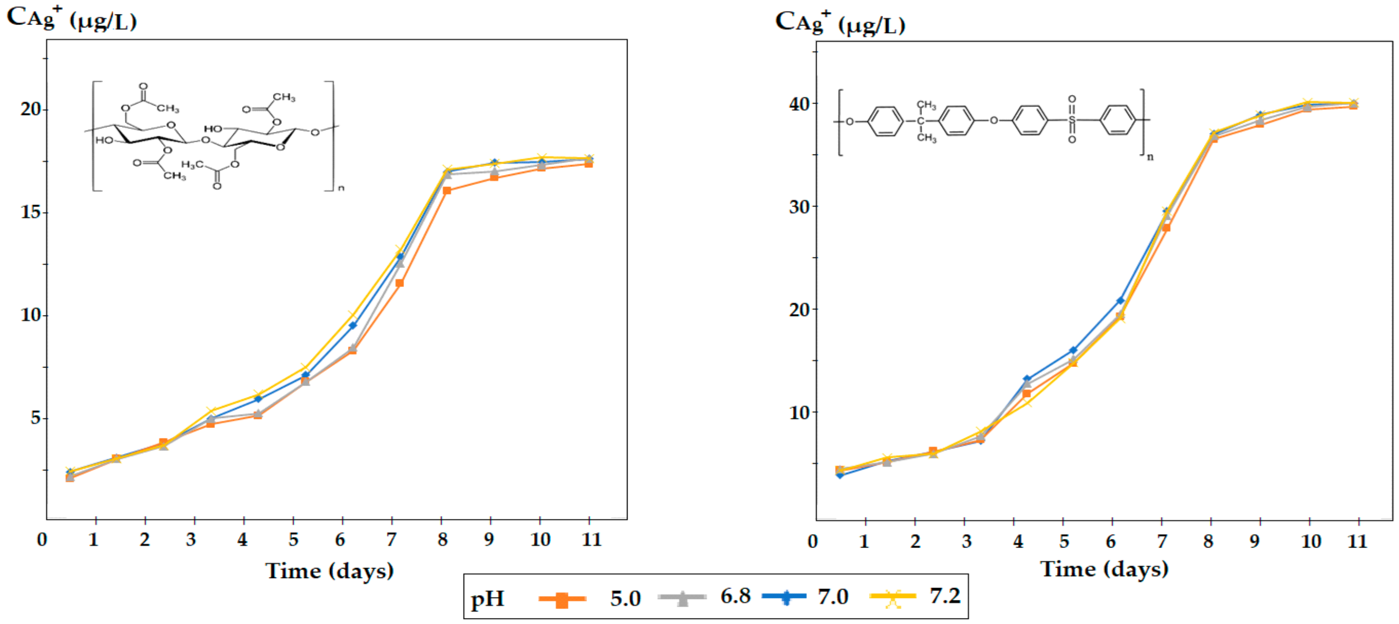

Figure S2. Concentration of silver ions released in the receiving aqueous phase of pH: 5.0; 6.8; 7.0; and 7.2 for cellulose acetate and polysulfone membrane in the case of 10–undecen–1–ol as dispersant and oxide nanoparticles with 1.63% silver.

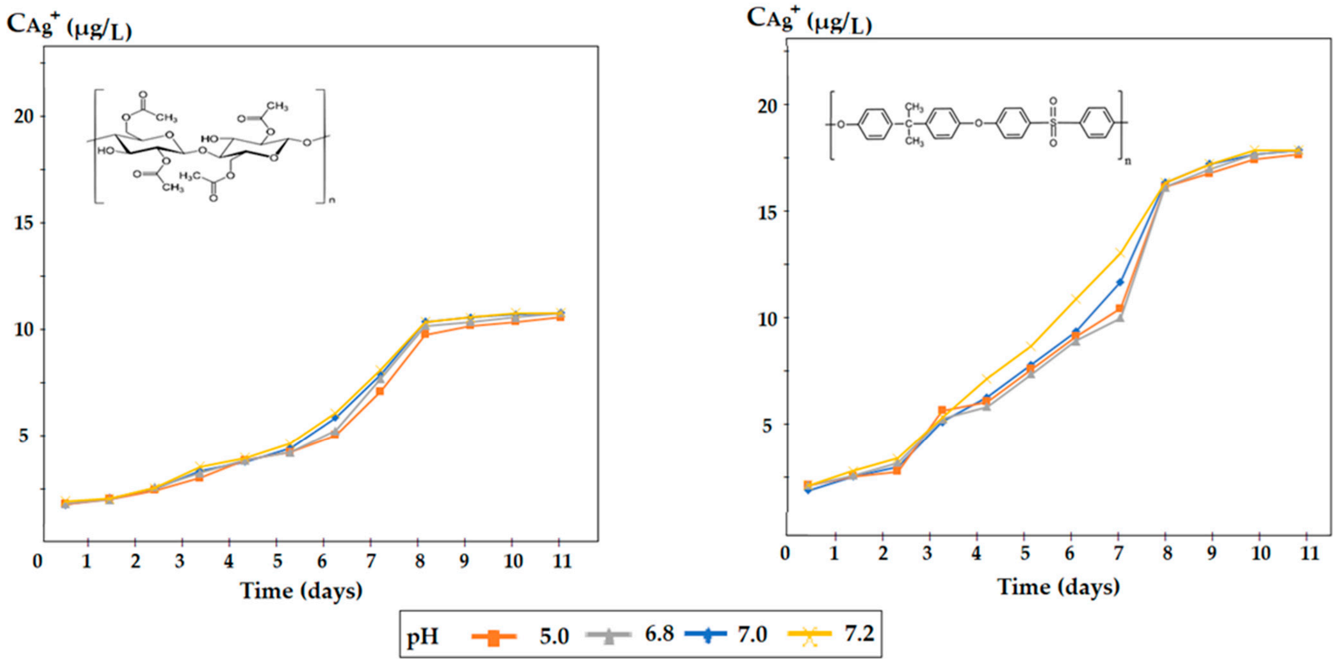

Figure S3. Concentration of silver ions released in the receiving aqueous phase of pH: 5.0; 6.8; 7.0; and 7.2 for cellulose acetate and polysulfone membrane in the case of 10–undecenoic chloride as dispersant and oxide nanoparticles with 1.63% silver.

It is interesting that 10–undecenol chloride, being insoluble in water, does not favour the transfer of silver ions in the aqueous receiving solution.

2. Influence of membrane support morphology and silver content of oxide nanoparticles on the release of silver ions in aqueous solution

For the polysulfone support membrane matrix, which provided the highest concentrations of released silver ions, the effect of macro-porous surface morphology (Figure S4) on the release process using the three types of oxide nanoparticles was studied, containing: 0.55%; 1.12%; and 1.63% silver, respectively.

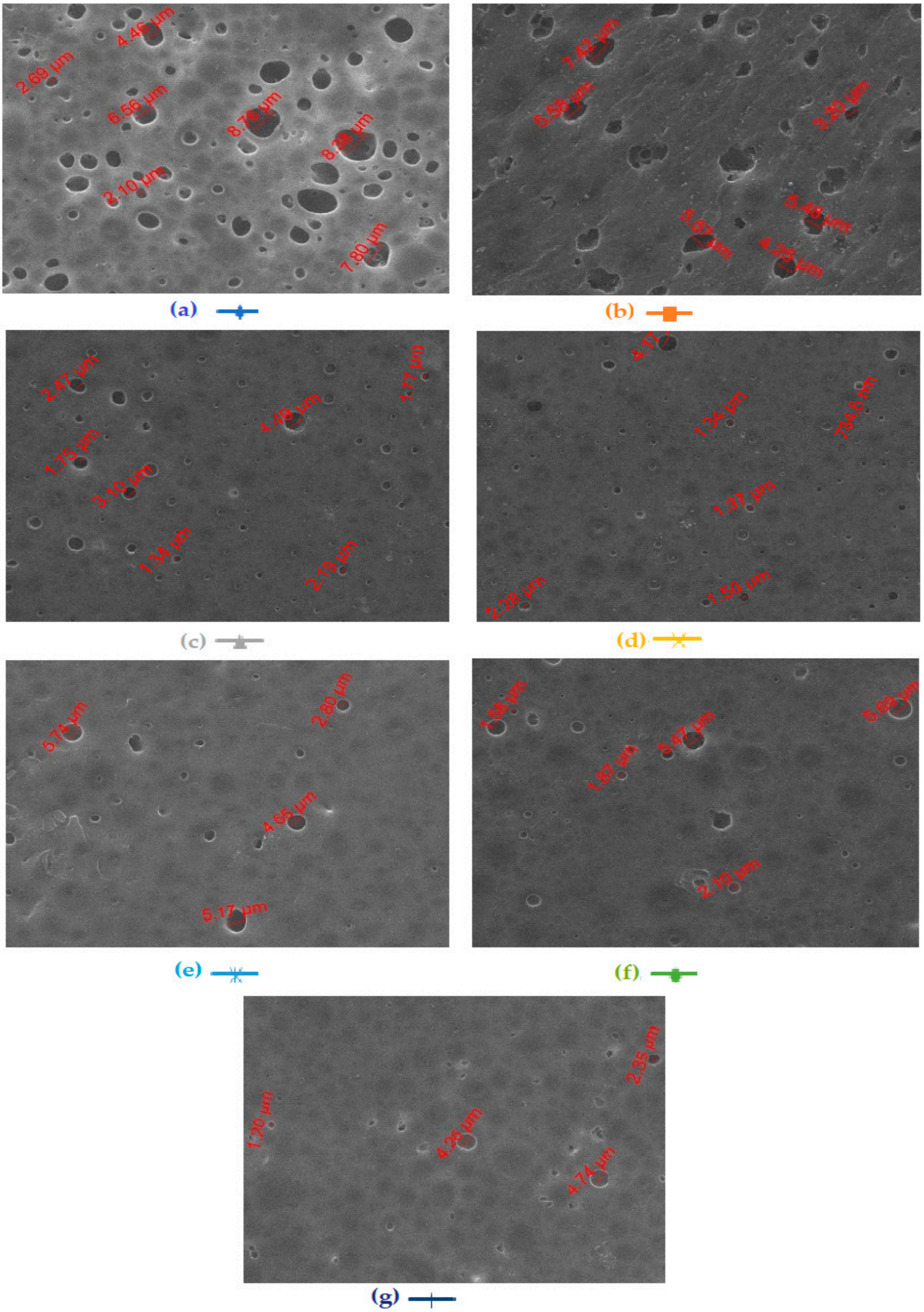

Figure S4. Morphology of polysulfone support membranes obtained by different exposure to the polymeric film before coagulation.
